# Mapping the Positive and Negative Syndrome Scale scores to EQ-5D-5L and SF-6D utility scores in patients with schizophrenia

**DOI:** 10.1007/s11136-018-2037-7

**Published:** 2018-10-31

**Authors:** Edimansyah Abdin, Siow Ann Chong, Esmond Seow, Swapna Verma, Kelvin Bryan Tan, Mythily Subramaniam

**Affiliations:** 1 Research Division, Institute of Mental Health, Singapore, Singapore; 2 Department of Early Psychosis Intervention, Institute of Mental Health, Singapore, Singapore; 3 Policy Research and Evaluation Division, Ministry of Health, Singapore, Singapore

**Keywords:** PANSS, Mapping, EQ-5D, SF-6D, Prediction, Schizophrenia

## Abstract

**Objective:**

The current study aims to map the Positive and Negative Syndrome Scale (PANSS) onto the five-level EuroQol five-dimensional (EQ-5D-5L) and Short Form six-dimensional (SF-6D) utility scores for patients with schizophrenia.

**Methods:**

A total of 239 participants with schizophrenia spectrum disorder were recruited from a tertiary psychiatric hospital in Singapore. Ordinary least squares (OLS), censored least absolute deviations and Tobit regression methods were employed to estimate utility scores from the EQ-5D-5L and SF-6D. Model selection of the 18 regression models (three regression methods × six model specifications) was primarily determined by the smallest mean absolute error and mean square error, and the largest *R*^2^ and adjusted *R*^2^.

**Results:**

The mean age of the sample was 39.7 years (SD = 10.3). The mean EQ-5D-5L and SF-6D utility scores were 0.81 and 0.68, respectively. The EQ-5D-5L utility scores were best predicted by the OLS regression model consisting of three PANSS subscales, i.e. positive, negative and general psychopathology symptoms, and covariates including age and gender. The SF-6D was best predicted by OLS regression model consisting of five PANSS subscales, i.e. positive, negative, excitement, depression and cognitive subscales.

**Conclusion:**

The current study provides important evidence to clinicians and researchers on mapping algorithms for converting PANSS scores into utility scores that can be easily applicable for cost–utility analysis when EQ-5D-5L and SF-6D data are not available for patients with schizophrenia spectrum disorder in Singapore.

**Electronic supplementary material:**

The online version of this article (10.1007/s11136-018-2037-7) contains supplementary material, which is available to authorized users.

## Introduction

Schizophrenia is a severe mental disorder which is highly disabling in nature and results in substantial costs to the patient and their family members [1]. The global annual cost of the schizophrenia varies between countries and ranged from US$94 million in Puerto Rico to US$102 billion in the US in 2013 [2]. Although a wide range of interventions have been introduced for the care and treatment of people with schizophrenia, due to scarce healthcare resources, cost–utility analyses have been increasingly used to inform decision making on appropriate resource allocation for interventions for the care and treatment of people with schizophrenia [3]. The quality-adjusted life-year (QALY) is an important outcome measure in cost–utility analyses as it combines both quality and quantity of life into a single measure which allows a broader comparison not only across treatment strategies but also across patient populations [4, 5]. Generic preference-based measures, such as the EuroQoL five-dimensional (EQ-5D) and the Short Form-6D (SF-6D) [5–7] are often recommended to estimate QALY for cost–utility analyses.

In clinical populations, however, the generic preference-based measures are not used as often as clinical instruments. In the absence of generic preference-based instruments, mapping is a useful tool and can be used as an alternative solution to estimate utility scores from clinical instruments [5–7]. This technique is called ‘‘map’’, or “crosswalk”, as it can produce statistical formulas or algorithms that allow a disease-specific or clinical instrument to predict utility scores from generic preference-based measures and subsequently generate QALY for cost–utility analyses in clinical studies [5, 8]. A systematic review has identified 144 studies mapping 110 different source instruments to EQ-5D and it was suggested that the number of mapping studies will continue to increase in the future [9]. However, we found that there are few mapping studies among patients with schizophrenia. To our knowledge only one study has been conducted so far to map Positive and Negative Syndrome (PANSS) scores onto EQ-5D and Short Form six-dimensional (SF-6D) utility scores using the direct method in the schizophrenia sample [10]. Findings showed that EQ-5D scores were best predicted by age, gender, general psychopathology and depressive symptoms [10].

The PANSS [11] is one of the most widely used clinical instruments to measure symptom severity of schizophrenia in clinical settings. It should be noted that the previous study [10] used a linear regression or ordinary least square (OLS) model to map utility scores from three PANSS factors (e.g. positive, negative and general psychopathology symptoms). It was reported that the performance of other alternative factor structure of the PANSS such as five-factor model [12] may be more appropriate for an Asian sample. There is also a growing literature which suggests that OLS model is unable to capture the EQ-5D score distribution which is often skewed and has a larger ceiling effect at value of 1. Given that limited data exist on mapping studies using the PANSS in Asian schizophrenia samples, further research is needed to understand how a mapping study using a different PANSS factor structure and statistical methods actualises in this population. Singapore is an island city-state in Southeast Asia, with a multi-ethnic Asian population of approximately 5.61 million people in 2016. The population comprises Chinese (74.3%), Malays (13.4%), Indians (9.1%) and other ethnic groups (3.2%) [13]. Thus, a mapping study done in Singapore can provide findings which can be extrapolated to other Asian populations with schizophrenia disorders. Hence, the current study aimed to map the PANSS onto the EQ-5D and SF-6D to inform future cost–utility analyses for treatment of schizophrenia in a multi-ethnic Asian sample.

## Methods

This is a cross-sectional study that aimed to study generic preference-based measures of health-related quality of life in patients with schizophrenia and depression. The study was conducted at the Institute of Mental Health (IMH) in Singapore between August 2016 and November 2017. IMH is the national tertiary psychiatric care provider which serves a large number of patients with diverse mental needs in Singapore. Participants were patients recruited from outpatient clinics at IMH. Inclusion criteria comprised patients who were Singapore citizens or permanent residents, aged 21 years and above, able to understand and speak English and having a clinical diagnosis of schizophrenia spectrum disorder. Patients who were incapable of doing the interview due to severe physical or mental illnesses and aged less than 21 years were excluded from the study. Prior to the commencement of the study, written informed consent was obtained from all study participants. The study was approved by the relevant institutional ethics review board (National Healthcare Group Domain Specific Review Board). For the purpose of the current study, data on socio-demographic background, EQ-5D-5L, SF-36 and PANSS from 251 participants were included. After removing observations with missing values in key variables, 239 observations were included in the final sample for analysis.

### Measures

1. The EQ-5D-5L comprises five items/dimensions on mobility, self-care, pain/discomfort, usual activities, and anxiety/depression with five possible answers for each item (1 = no problems, 2 = slight problems, 3 = moderate problems, 4 = severe problems, 5 = extreme problems) and can generate 3125 possible health states. The utility scores of EQ-5D-5L were obtained using the UK value set estimated using a crosswalk approach. The crosswalk approach was developed by van Hout et al. [14] using the crosswalk link function between the EQ-5D-3L value sets and the new EQ-5D-5L descriptive system.
2. The SF-6D is a multidimensional health classification system assessing the six health domains of physical functioning, role limitation, social functioning, pain, mental health and vitality, with 4–6 levels for each domain derived from 11 items of the Short Form 36 item questionnaire. The utility scores of SF-6D were obtained using the UK value set estimated using a SF-6D scoring algorithm. The SF-6D scoring algorithm was developed using the standard gamble (SG) method from a sample of 249 SF-6D health states from a representative sample of the UK population [15]. A previous study has found that the utility scores derived from English and Chinese versions of the SF-6D have been demonstrated to be equivalent in Singapore [16].
3. The PANSS [11] is a 30-item instrument designed to measure the severity of three dimensions of symptoms [positive (7 items), negative (7 items) and general psychopathology (16 items)] among those with schizophrenia spectrum disorder. The symptom severity was assessed by a trained interviewer following a semi-structured interview with the participant. Each symptom was rated on a seven-point scale representing increasing levels of psychopathology (1 = absent to 7 = extreme) with total scores ranging from 30 to 210. The PANSS total score and the three-factor scores including positive (scores ranging from 7 to 49), negative (scores ranging from 7 to 49) and general psychopathology (scores ranging from 16 to 112) dimensions were obtained by adding scores of the respective items in each subscale [11]. A previous study [12] in our local population found that PANSS could be further divided into five factors and reduced into 17 items: positive (scores ranging from 4 to 28), negative (scores ranging from 5 to 35), excitement (scores ranging from 3 to 21), depression (scores ranging from 3 to 21) and cognitive (scores ranging from 2 to 14) factors. The construct validity of five-factor structure has been validated in Singapore [12]. Hence, the five-factor structure of PANSS was also tested in the current study.

### Statistical analyses

Statistical analyses were carried out using the STATA software version 13 (StataCorp LP, College Station, TX). Since the distribution of utility scores derived from generic preference-based measures such as EQ-5D are often not normally distributed and have higher ceiling effect at value of 1 [17], we decided to use three regression methods including the OLS, censored least absolute deviations (CLAD) [18] and Tobit [19] regression models to predict utility scores from the PANSS. The selection of these regression methods was based on their frequency of use and applicability to estimate the utility scores [5, 20–23]. The OLS (Eq. 1) is the most widely used regression method which can be expressed as

1 $$Y_{{\text{i}}} = \beta _{0} + \beta _{1} X_{{1{\text{i}}}} + \cdots + \beta _{{\text{k}}} X_{{k{\text{i}}}} + \varepsilon _{{\text{i}}} ,$$

where ${ Y}_{i}$ is the utility score for subject *i*, ${\beta }_{0}$ is the intercept, ${\beta }_{1},\dots .{\beta }_{k}$ are the regression coefficients (slopes), ${X}_{1i},\dots {X}_{ki}$ are the independent variables including PANSS total score, PANSS factor scores, age and gender and ${\varepsilon }_{i}$ is the error term. In the OLS model, the slopes and intercept were estimated by minimising the sum of the squares of the differences between the observed and predicted utility scores. This model assumes that the errors ${\varepsilon }_{i}$ are normally distributed with mean zero and constant variance (homoscedasticity) as denoted by ${\varepsilon }_{i}=N(0,{\sigma }^{2})$.

2 $${\text{Tobit: }}Y_{i}^{*} = \beta _{0} + \beta _{1} X_{1} i + \cdots + \beta _{k} X_{k} i + \varepsilon _{i}$$

The Tobit model (Eq. 2) is a regression model used in the presence of censored data which assumes that if a patient’s observed EQ-5D utility score is 1, then ${Y}_{i}^{*}$ is greater than 1 (Eq. 3). It means despite having the same observed score at the ceiling of 1, patients with these responses may be different and that their true health state may vary [19, 24, 25]. This model assumes that there is a latent utility score ${Y}_{i}^{*}$ that represents a valuation of an individual’s true health state. Hence, it is the latent utility score ${ Y}_{i}^{*}$, rather than the observed utility score ${ Y}_{i}$ was modelled.

3 $${Y_i}=Y_{i}^{*}{\text{ for }}Y_{i}^{*}<1\,\,{\text{and}}\,\,{Y_i}=1\,\,{\text{for }}\,Y_{i}^{*}>1.$$

Similar to Tobit model (Eq. 3), the CLAD model assumes that the EQ-5D utility score of 1 has been censored and therefore the latent utility ${Y}_{i}^{*}$ is modelled. However, in contrast to OLS and Tobit model, the CLAD model regresses the median of the latent utility ${Y}_{i}^{*}$ instead of the mean and minimises the sum of absolute deviations instead of minimising the sum of squares of the differences between the observed and predicted utility scores to estimate the regression slopes [26].

Six different model specifications were tested in each regression method after taking into account total score, the three original factor scores and the five-factor model of the PANSS that was proposed for Asian samples [12] as well as recent findings from a mapping study by Siani et al. [10]. The model specifications are outlined in detail in Table 1. Model 1 included only PANSS total score as a main predictor for the utility score; Model 2 included PANSS positive, negative, and general psychopathology symptom scores; Model 3 included PANSS positive, negative, excitement, depression and cognitive scores; Model 4 included PANSS total score, age and gender; Model 5 included PANSS positive, negative, general psychopathology symptom scores, age and gender; Model 6 included PANSS positive, negative, excitement, depression, cognitive scores, age and gender. These similar model specifications were also tested for the SF-6D utility score using OLS, CLAD and Tobit regression models. A number of posteriori specification tests including normality, multicollinearity and homoscedasticity assumptions were conducted to validate the final regression model [27].

**Table 1:** Model specifications

Model 1	OLS	$= \beta _{0} + \beta _{1} PANSStotal_{{\text{i}}} + \varepsilon _{i}$
	Tobit	$= \beta _{0} + \beta _{1} PANSStotal_{{\text{i}}} + \varepsilon _{i}$
	CLAD	$={\beta }_{0}+{\beta }_{1}{\text{PANSStotal}}_{\text{i}}+{\varepsilon }_{i}$
Model 2	OLS	$= \beta _{0} + \beta _{1} PANSSpositive_{{\text{i}}} + \beta _{2} PANSSnegative_{{\text{i}}} + \beta _{3} PANSSgeneralpsychopathology_{i} + e_{i}$
	Tobit	$= \beta _{0} + \beta _{1} PANSSpositive_{{\text{i}}} + \beta _{2} PANSSnegative_{{\text{i}}} + \beta _{3} PANSSgeneralpsychopathology_{i} + e_{i}$
	CLAD	$={\beta }_{0}+{\beta }_{1}{PANSS positive}_{i}+{\beta }_{2}{PANSS negative}_{i}+{\beta }_{3}{PANSSgeneralpsychopathology}_{i}+{e}_{i}$
Model 3	OLS	$= \beta _{0} + \beta _{1} PANSSpositive_{{\text{i}}} + \beta _{2} PANSSnegative_{i} + \beta _{3} PANSSexcitement_{{\text{i}}}$ $+ \beta _{4} PANSSdepression_{{\text{i}}} + \beta _{5} PANSScognitive_{{\text{i}}} + e_{i}$
	Tobit	$= \beta _{0} + \beta _{1} PANSSpositive_{{\text{i}}} + \beta _{2} PANSSnegative_{{\text{i}}} + \beta _{3} PANSSexcitement_{{\text{i}}}$ $+ \beta _{4} PANSSdepression_{{\text{i}}} + \beta _{5} PANSScognitive_{{\text{i}}} + e_{i}$
	CLAD	$={\beta }_{0}+{\beta }_{1}{PANSS positive}_{i}+{\beta }_{2}{PANSS negative}_{i}+{\beta }_{3}{PANSS excitement}_{i}$ $+{ \beta }_{4}{PANSS depression}_{i}+{\beta }_{5}{PANSS cognitive}_{i}+{e}_{i}$
Model 4	OLS	$={\beta }_{0}+{\beta }_{1}{PANSS total}_{i}+{\beta }_{2}{age}_{i}+{\beta }_{3}{gender}_{i}+{\varepsilon }_{i}$
	Tobit	$={\beta }_{0}+{\beta }_{1}{PANSS total}_{i}+{\beta }_{2}{age}_{i}+{\beta }_{3}{gender}_{i}+{\varepsilon }_{i}$
	CLAD	$={\beta }_{0}+{\beta }_{1}{PANSS total}_{i}+{\beta }_{2}{age}_{i}+{\beta }_{3}{gender}_{i}+{\varepsilon }_{i}$
Model 5	OLS	$={\beta }_{0}+{\beta }_{1}{PANSS positive}_{i}+{\beta }_{2}{PANSS negative}_{i}+{\beta }_{3}{PANSSgeneralpsychopathology}_{i}$ $+{ \beta }_{4}{age}_{i}+{\beta }_{5}{gender}_{i}+{\varepsilon }_{i}$
	Tobit	$={\beta }_{0}+{\beta }_{1}{PANSS positive}_{i}+{\beta }_{2}{PANSS negative}_{i}+{\beta }_{3}{PANSSgeneralpsychopathology}_{i}$ $+{ \beta }_{4}{age}_{i}+{\beta }_{5}{gender}_{i}+{\varepsilon }_{i}$
	CLAD	$={\beta }_{0}+{\beta }_{1}{PANSS positive}_{i}+{\beta }_{2}{PANSS negative}_{i}+{\beta }_{3}{PANSSgeneralpsychopathology}_{i}$ $+{ \beta }_{4}{age}_{i}+{\beta }_{5}{gender}_{i}+{\varepsilon }_{i}$
Model 6	OLS	$={\beta }_{0}+{\beta }_{1}{PANSS positive}_{i}+{\beta }_{2}{PANSS negative}_{i}+{\beta }_{3}{PANSS excitement}_{i}$ $+{ \beta }_{4}{PANSS depression}_{i}+{\beta }_{5}{PANSS cognitive}_{i}+{ \beta }_{6}{age}_{i}+{\beta }_{7}{gender}_{i}+{\varepsilon }_{i}$
	Tobit	$={\beta }_{0}+{\beta }_{1}{PANSS positive}_{i}+{\beta }_{2}{PANSS negative}_{i}+{\beta }_{3}{PANSS excitement}_{i}$ $+{ \beta }_{4}{PANSS depression}_{i}+{\beta }_{5}{PANSS cognitive}_{i}+{ \beta }_{6}{age}_{i}+{\beta }_{7}{gender}_{i}+{\varepsilon }_{i}$
	CLAD	$={\beta }_{0}+{\beta }_{1}{PANSS positive}_{i}+{\beta }_{2}{PANSS negative}_{i}+{\beta }_{3}{PANSS excitement}_{i}$ $+{ \beta }_{4}{PANSS depression}_{i}+{\beta }_{5}{PANSS cognitive}_{i}+{ \beta }_{6}{age}_{i}+{\beta }_{7}{gender}_{i}+{\varepsilon }_{i}$

The best fit model of the 18 regression models (three regression methods X six model modifications) (Table 1) was assessed based on the four goodness-of-fit indices [29] including mean absolute error (MAE)—the mean of the absolute differences between observed and the predicted utility scores; mean square error (MSE)—the average of the squared differences between the observed and the predicted utility scores; *R*^2^ and adjusted *R*^2^ [7]. With *R*^2^ and adjusted *R*^2^ values, the higher the value, the better the model, and with MAE and MSE values, the lower the value, the better the model fit. The coefficient of determination, *R*^2^ and adjusted *R*^2^ parameters derived from OLS regression model were not compatible across regression methods as the *R*^2^ from OLS regression model was based on coefficient of determination between the observed and the predicted scores, while *R*^2^ from the CLAD and Tobit regression model were calculated based on likelihood ratio between the intercept-only model and the full model [23, 28]. For purposes of fair comparison, the *R*^2^ from three regression methods (OLS, CLAD and Tobit) were calculated by squaring the correlation coefficient of the observed and the predicted utility scores. Adjusted *R*^2^ was computed using the following formula after penalising the complexity model [23]:

$${\text{Adjusted }}{R^2}=1 - \frac{{(n - 1)}}{{(n - p - 1)}}(1 - {R^2}),$$

where *n* is the sample size and *p* is the number of parameters in the model.

Lastly, the distributions of the observed and predicted utility values in terms of mean and standard deviation were also compared across models to guide selection of the best prediction model.

## Results

### Descriptive statistics

**Fig. 1:**
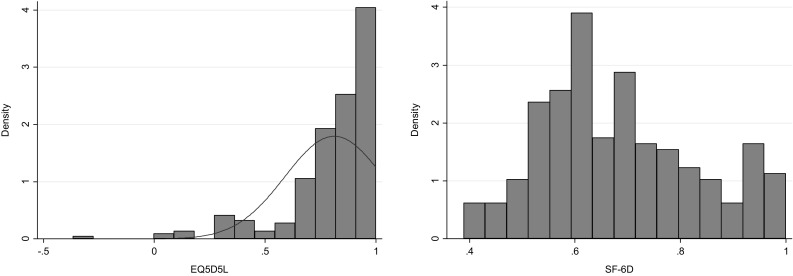
Observed EQ-5D-5L and SF-6D utility scores

The descriptive statistics are presented in Table 2. The sample included 239 participants with schizophrenia spectrum disorder. The mean age of the overall sample was 39.7 years (SD = 10.3), 59.8% were Chinese, 19.3% were Malays, 18.4% were Indians and 2.5% belonged to other ethnicities. The EQ-5D-5L showed a mean (SD) index score of 0.81 (0.2) with minimum and maximum scores of − 0.367 and 1 while the mean (SD) SF-6D index was 0.68 (0.15) with minimum and maximum scores of 0.389 and 1, respectively. An inspection of the distribution of the EQ-5D-5L scores showed a substantial skew to the right, that is, towards better quality of life (Fig. 1). The mean (SD) PANSS total score and its three factors including positive, negative and general psychopathology symptoms were 47.8 (15.4), 12.1 (5.5), 10.8 (5.0) and 24.9 (7.9), respectively. The mean (SD) PANSS five-factor scores including positive, negative, excitement, depression and cognitive factors were 8.1 (5.0), 7.5 (3.6), 4.3 (2.0), 6.1 (3.3) and 2.9 (1.5), respectively.

**Table 2:** Characteristics of the sample

	*N* (%)
Age, mean (SD)	39.70 (10.28)
Gender	
Female	105 (43.9)
Male	134 (56.1)
Ethnicity	
Chinese	143 (59.8)
Malay	46 (19.3)
Indian	44 (18.4)
Others	6 (2.5)
Education	
Primary and below	25 (10.4)
Secondary	91 (30.1)
Post secondary to Pre-University	86 (36.0)
University	37 (15.5)
Marital status	
Never married	180 (75.3)
Currently married	31 (13.0)
Separated	9 (3.8)
Divorced	17 (7.1)
Widowed	2 (0.8)

### Mapping on EQ-5D-5L

Table 3 shows regression coefficients and goodness-of-fit measures of the three regression methods (OLS, CLAD and Tobit) for mapping PANSS to the EQ-5D-5L and SF-6D utility scores. Among the three regression methods, OLS generally had the largest *R*^2^ and adjusted *R*^2^, and smallest MSE, regardless of the model specifications. For each regression method, six model specifications were fitted. We found model 5 consisting of the positive, negative, general psychopathology symptoms, age and gender had the largest adjusted *R*^2^, and smallest MSE. The model explained 33.8% of the variation with minimal MSE (0.0328) and MAE (0.1348), respectively. A histogram used to examine the normality assumption of the final model showed that the distribution of the residuals was approximately normal (Supplementary Fig. 1). Possible multicollinearity problem between predictors were determined by obtaining the variance inflation factor (VIF). If the VIF value was more than 10, multicollinearity was considered. No significant multicollinearity effect was observed between EQ-5D predictors (VIF values ranging from 1.00 to 2.53) (Supplementary Table 1). The Breusch–Pagan (BP) test was used to detect heteroscedasticity. If homoscedasticity assumption was rejected, heteroscedasticity robust standard error adjustment based on Huber–White sandwich estimator of the variance was used for inference [27]. The BP test statistic showed that the null hypothesis of homoscedasticity assumption of the model was rejected (Chi-square (degree of freedom): 46.5(5), *p* value < 0.001). Therefore, heteroscedasticity robust standard error adjustment was used for inference. In this final model, the EQ-5D-5L utility values could be generated using the following mapping algorithm for schizophrenia sample in the absence of EQ-5D data:

$${\text{EQ-5D-5L utility}} = {\text{1}}.{\text{31}}0{\text{3}} - 0.00{\text{44 }} \times {\text{ positive}} + 0.00{\text{25 }} \times {\text{ negative}} - 0.0{\text{146 }} \times {\text{ generalpsychopathology}} - 0.00{\text{29 }} \times {\text{ age}} + 0.0{\text{149 }} \times {\text{ female}}.$$

**Table 3:** Regression coefficients and goodness-of-fit measures of three regression methods for mapping PANSS to the EQ-5D-5L and SF-6D utility scores

	EQ-5D-5L			SF-6D		
	OLS	CLAD	Tobit	OLS	CLAD	Tobit
Model specification						
Model 1						
PANSS total	− 0.0078*	− 0.0076*	− 0.0105*	− 0.0044*	− 0.0038*	− 0.0045*
Goodness-of-fit indices						
*R*^2^	0.2890	0.2890	0.2890	0.2072	0.2072	0.2072
Adjusted *R*^2^	0.2860	0.2860	0.2860	0.2039	0.2039	0.2039
MSE	0.0353	0.0371	0.0427	0.0177	0.0179	0.0177
MAE	0.1425	0.1382	0.1542	0.1102	0.1100	0.1105
Model 2						
Positive	− 0.0043	− 0.0037	− 0.0066	− 0.0026	− 0.0014	− 0.0027
Negative	0.0023	0.0053	0.0030	0.00004	− 0.0001	0.00001
General psychopathology	− 0.0146*	− 0.0169*	− 0.0191*	− 0.0076*	0.0079*	− 0.0078*
Goodness-of-fit indices						
*R*^2^	0.3327	0.3220	0.3325	0.2263	0.2200	0.2263
Adjusted *R*^2^	0.3242	0.3134	0.3240	0.2164	0.2100	0.2164
MSE	0.0334	0.0358	0.0408	0.0174	0.0180	0.0174
MAE	0.1359	0.1312	0.1481	0.2000	0.1095	0.1107
Model 3						
Positive	− 0.0100*	− 0.0100*	− 0.0140*	− 0.0057*	− 0.0042	− 0.0058*
Negative	− 0.0119*	− 0.0045	− 0.0165*	− 0.0076*	− 0.0084*	− 0.0077*
Excitement	− 0.0038	− 0.0083	− 0.0035	− 0.0050	− 0.0014	− 0.0054
Depression	− 0.0223*	− 0.0247*	− 0.0322*	− 0.0149*	− 0.0137*	− 0.0153*
Cognitive	− 0.0023	0.0008	0.0052	− 0.0100	− 0.0094	0.0103
Goodness-of-fit indices						
*R*^2^	0.3244	0.3054	0.3295	0.2869	0.2564	0.2869
Adjusted *R*^2^	0.3099	0.2905	0.3151	0.2716	0.2405	0.2716
MSE	0.0338	0.0371	0.0420	0.0162	0.0170	0.0162
MAE	0.1388	0.1306	0.1540	0.1056	0.1069	0.1059
Model 4						
PANSS total	− 0.0160*	− 0.0064*	− 0.0105*	− 0.0088*	− 0.0042*	− 0.0045*
Age	− 0.0029*	− 0.0034*	− 0.0045*	− 0.0005	− 0.0019	− 0.0004
Gender	0.0091	0.0295	0.0289	0.0012	0.0128	0.0029
Goodness-of-fit indices						
*R*^2^	0.3430	0.3015	0.3064	0.2230	0.1662	0.2085
Adjusted *R*^2^	0.3346	0.2926	0.2975	0.2130	0.1556	0.1984
MSE	0.0329	0.0374	0.0426	0.0175	0.0190	0.0178
MAE	0.1359	0.1377	0.1579	0.1107	0.1105	0.1111
Model 5						
Positive	− 0.0044	− 0.0071*	− 0.0070	− 0.0026	− 0.0027	− 0.0027
Negative	0.0025	0.0062*	0.0033	− 0.00005	− 0.0005	0.0001
General psychopathology	− 0.0146*	− 0.125*	− 0.0189*	− 0.0075*	− 0.0072*	− 0.0077*
Age	− 0.0029*	− 0.0024*	− 0.0045*	− 0.0005	− 0.0014	− 0.0004
Gender	0.0149	0.0332	0.0338	0.0031	0.0109	0.0041
Goodness-of-fit indices						
*R*^2^	0.3519	0.2922	0.3509	0.2278	0.2083	0.2277
Adjusted *R*^2^	0.3380	0.2770	0.3370	0.2112	0.1913	0.2111
MSE	0.0328	0.0385	0.0406	0.0175	0.0192	0.0175
MAE	0.1348	0.1322	0.1513	0.1109	0.1146	0.1112
Model 6						
Positive	− 0.0096*	− 0.0105*	− 0.0137*	− 0.0056*	− 0.0054*	− 0.0057*
Negative	− 0.0122*	− 0.0051	− 0.0170*	− 0.0078*	− 0.0092*	− 0.0079*
Excitement	− 0.0045	− 0.0084	− 0.0045	− 0.0052	− 0.0026	− 0.0055
Depression	− 0.0227*	− 0.0230*	− 0.0323*	− 0.0152*	− 0.0138*	− 0.0155*
Cognitive	− 0.0018	0.0018	0.0064	− 0.0102	0.0111	0.0105
Age	− 0.0032*	− 0.0013	− 0.0050*	− 0.0008	− 0.0016	− 0.0007
Gender	0.0082	− 0.0038	0.0220	0.0033	0.0093	0.0025
Goodness-of-fit indices						
*R*^2^	0.3539	0.3008	0.3512	0.2900	0.2639	0.2899
Adjusted *R*^2^	0.3343	0.2780	0.3315	0.2685	0.2416	0.2684
MSE	0.0329	0.0376	0.0417	0.0162	0.0170	0.0162
MAE	0.1372	0.1369	0.1581	0.1058	0.1048	0.1061

*p value < 0.05

The model revealed that general psychopathology symptoms and age were significantly and inversely associated with EQ-5D-5L utility scores. The observed and predicted EQ-5D-5L and SF-6D utility scores by six different model specifications are compared in Table 3. It reveals that the means of the predicted values based on OLS were similar to the observed EQ-5D-5L values, while the means of the predicted values based on CLAD and Tobit models tended to produce larger predicted values than the observed values (Table 4).

**Table 4:** Descriptive statistics of the observed and predicted utility scores by OLS, CLAD and Tobit models

	EQ-5D-5L		SF-6D	
	Mean	SD	Mean	SD
Observed EQ-5D-5L utility scores	0.8117	0.2225	0.6836	0.1490
Predicted EQ-5D-5L utility scores				
OLS				
Model 1	0.8117	0.1196	0.6836	0.0678
Model 2	0.8117	0.1283	0.6836	0.0709
Model 3	0.8117	0.1281	0.6836	0.0798
Model 4	0.8117	0.1303	0.6836	0.0704
Model 5	0.8117	0.1320	0.6836	0.0711
Model 6	0.8117	0.1323	0.6836	0.0802
CLAD				
Model 1	0.8514	0.1364	0.6713	0.0745
Model 2	0.8474	0.1428	0.6633	0.0665
Model 3	0.8417	0.1502	0.6806	0.0622
Model 4	0.8372	0.1027	0.6671	0.0589
Model 5	0.8492	0.1253	0.6972	0.1001
Model 6	0.8667	0.1131	0.6708	0.0745
Tobit				
Model 1	0.8858	0.1626	0.6858	0.0696
Model 2	0.8843	0.1724	0.6858	0.0728
Model 3	0.8860	0.1769	0.6857	0.0817
Model 4	0.8860	0.1702	0.6858	0.0697
Model 5	0.8844	0.1795	0.6858	0.0729
Model 6	0.8862	0.1851	0.6857	0.0820

### Mapping on SF-6D

Among the three regression methods, OLS generally had slightly larger *R*^2^ and adjusted *R*^2^, and smaller MSE and MAE than the CLAD and Tobit regression methods. For each regression method, six model specifications were also fitted. We found model 3 consisting of the positive, negative, excitement, depression and cognitive factors had the largest adjusted *R*^2^, and smallest MSE and MAE than other model specifications. The distribution of the residuals was approximately normal (Supplementary Fig. 1). No significant multicollinearity effect was observed between SF-6D predictors (VIF values were ranged from 1.17 to 1.53) (Supplementary Table 1). However, BP test statistic showed that the null hypothesis of homoscedasticity assumption of the model was rejected (Chi-square (degree of freedom): 17(5), *p* value = 0.003). Therefore, heteroscedasticity robust standard error adjustment was used for inference. This model explained 27.2% of the variation with minimal MSE (0.0162) and MAE (0.1056), respectively. Hence, the SF-6D utility scores could be generated using the following mapping algorithm:

$${\text{SF-6D utility}} = 0.{\text{8}}7{\text{12}} - 0.00{\text{57 }} \times {\text{ positive}} - 0.00{\text{76 }} \times {\text{ negative}} - 0.00{\text{5}}0{\text{ }} \times {\text{ excitement}} - 0.0{\text{149 }} \times {\text{ depression}} + 0.0{\text{1}}00{\text{ }} \times {\text{ cognitive}}.$$

In this final model, positive, negative and depression factor scores were significantly and inversely associated with SF-6D utility scores. The means of the predicted values based on OLS were similar to the observed EQ-5D-5L values. The means of the predicted values based on CLAD model tended to produce smaller predicted values than the observed values, while the means of the predicted values based on Tobit model tended to produce larger predicted values than the observed values (Table 4).

## Discussion

This is one of the few studies that has been conducted to map PANSS on two common utility scores, the EQ-5D-5L and SF-6D, in people with schizophrenia spectrum disorder in a multi-ethnic Asian population. In the current study, three different regression methods and 6 model specifications were explored to develop mapping functions for PANSS. The findings provide evidence that different predictive models should be used for mapping EQ-5D-5L and SF-6D in the Asian sample. Our regression analyses showed that the EQ-5D-5L utility scores of schizophrenia spectrum disorder patients in our sample was best predicted by the OLS model consisting of three PANSS factors, i.e. positive, negative and general psychopathology symptoms, and covariates including age and gender (Model 5). The final model explained 33.8% of the variation with minimal MSE (0.0328) and MAE (0.1348), respectively. Our mapping algorithm for SF-6D was best predicted by model 3 consisting of five PANSS factors, i.e. positive, negative, excitement, depression and cognitive. This model explained 27.2% of the variation with minimal MSE (0.0162) and MAE (0.1056), respectively. In predicting EQ-5D-5L utility scores, we note, however, that only PANSS general psychopathology symptoms and age were significantly and inversely associated with EQ-5D-5L utility scores. A previous study [10] has shown that the PANSS general psychopathology symptoms, age, gender and depressive symptoms as measured by Calgary Depression Scale for Schizophrenia (CDSS) were significantly associated with EQ-5D and SF-6D utility scores. Our results are not directly comparable with those of Siani et al. study [10] because we only included age and gender in the regression analyses. Apart from that, the differences in the findings between our study and the above study could be also due to the fact that the latter study had included CDSS scale in their regression model and the data were derived from European cohort studies. For this reason, we are unable to make a direct comparison with this study. However, it is important to note that the main purpose of the study was to develop a mapping function that best predicted utility scores derived from EQ-5D-5L and SF-6D, thus the statistical significance of the regression coefficients is of secondary consideration [23]. In the current study, model selection was primarily determined by four goodness-of-fit indices including *R*^2^, adjusted *R*^2^, MAE and MSE. Apart from that, the predictive ability of the model in terms of predicted mean scores was also taken into account in the model selection. Generally, our MAE values for the SF-6D were lower than MAE values (up to 0.15) that are typically reported in the literature [8]. The MAE values that were produced by OLS in our final model were slightly higher than that produced by CLAD model. Cheung et al. [23] have suggested that the MAE tends to favour the CLAD than the OLS model. Hence, the selection of the best model should not focus exclusively on one fit index but should take into consideration overall goodness-of-fit indices and descriptive statistics of the predicted scores. In the current study, the mean predicted EQ-5D-5L and SF-6D values at the group level based on OLS regression were similar to their mean observed values. These findings may support internal validity of the model and suggest that the mapping algorithm may be more appropriately used at a group level. Among the three regression methods, the means of the predicted values based on Tobit models tended to produce larger predicted values than the observed values. Previous studies have shown that the OLS was superior to Tobit as well as CLAD model [23, 28–30].

There are some limitations in the current study. First, the utility values for EQ-5D-5L were based on the crosswalk approach that mapped EQ-5D-5L utility scores from the EQ-5D-3L because the Singapore value set estimated from a valuation study has not yet been developed. Hence, results may have been different if the new value set had been used [31]. Second, the limited sample size did not allow us to test the model equally well in sub-samples of the overall sample. However, it should be noted that a recent set of guidelines issued by the ISPOR Good Practice for Outcomes Research Task Force has not recommended splitting the sample to validate results on part of the sample [32]. Hence, further validation of the current mapping findings using external dataset is recommended. Nonetheless, this is the first study to compare three regression methods to map a clinical instrument onto widely used generic preference-based measures specifically for schizophrenia spectrum disorder patients. The mapping process has incorporated a schizophrenia-specific clinical instrument and key demographic characteristics (i.e. age and gender) into the model which is feasible for use in economic evaluation of clinical research projects. From a clinical perspective, PANSS, age and gender are the most commonly used data to measure symptoms severity and characteristics of patients with schizophrenia either in trials or intervention programs in Singapore. For example, in Singapore’s Early Psychosis Intervention Programme’s (EPIP) [33] long-acting injectable risperidone (LAR) trial [34], information on symptom severity was routinely captured by case managers to monitor patients as well as to assess the efficacy of the antipsychotic medication but the trial lacked a cost-effectiveness component. The availability of this algorithm will make cost–utility analysis among patients with schizophrenia who are monitored only for symptom severity possible in future trials and program evaluation.

In conclusion, we have provided algorithms for converting PANSS scores into utility scores that is easily applicable in the clinical setting when EQ-5D and SF-6D data are not available. The current study provides important evidence to clinicians and researchers about the mapping algorithms that can be used for economic evaluation of patients with schizophrenia spectrum disorder in a multi-ethnic Asian patient population.

## Electronic supplementary material

Below is the link to the electronic supplementary material.

Supplementary material 1 (DOCX 37 KB)

## References

[1] WHO (1998). Schizophrenia and public health. Division of mental health and prevention of substance abuse.

[2] Chong HY, Teoh SL, Wu DB (2016). Global economic burden of schizophrenia: A systematic review. Neuropsychiatric Disease and Treatment.

[3] Andrew A, Knapp M, McCrone P (2012). Effective interventions in schizophrenia: The economic case, in personal social services research unit.

[4] Brazier J (2008). Measuring and valuing mental health for use in economic evaluation. The Journal of Health Services Research & Policy.

[5] Brazier J, Connell J, Papaioannou D (2014). A systematic review, psychometric analysis and qualitative assessment of generic preference-based measures of health in mental health populations and the estimation of mapping functions from widely used specific measures. Health Technology Assessment.

[6] NICE. (2013). *Guide to the methods of technology appraisal 2013, National Institute for Health and Care Excellence, UK*. https://www.nice.org.uk/process/pmg9/chapter/foreword. 27905712

[7] Longworth L, Yang Y, Young T (2014). Use of generic and condition-specific measures of health-related quality of life in NICE decision-making: A systematic review, statistical modelling and survey. Health Technology Assessment.

[8] Brazier JE, Yang Y, Tsuchiya A (2010). A review of studies mapping (or cross walking) non-preference based measures of health to generic preference-based measures. The European Journal of Health Economics.

[9] Dakin H, Abel L, Burns R (2018). Review and critical appraisal of studies mapping from quality of life or clinical measures to EQ-5D: An online database and application of the MAPS statement. Health and Quality of Life Outcomes.

[10] Siani C, de Peretti C, Millier A (2016). Predictive models to estimate utility from clinical questionnaires in schizophrenia: Findings from EuroSC. Quality of Life Research.

[11] Kay SR, Fiszbein A, Opler LA (1987). The positive and negative syndrome scale (PANSS) for schizophrenia. Schizophrenia Bulletin.

[12] Jiang J, Sim K, Lee J (2013). Validated five-factor model of positive and negative syndrome scale for schizophrenia in Chinese population. Schizophrenia Research.

[13] Singapore Department of Statistics (2017). Yearbook of statistics Singapore, 2017.

[14] van Hout B, Janssen MF, Feng YS (2012). Interim scoring for the EQ-5D-5L: Mapping the EQ-5D-5L to EQ-5D-3L value sets. Value in Health.

[15] Brazier J, Roberts J, Deverill M (2002). The estimation of a preference-based measure of health from the SF-36. Journal of Health Economics.

[16] Wee HL, Cheung YB, Fong KY (2004). Are English- and Chinese-language versions of the SF-6D equivalent? A comparison from a population-based study. Clinical Therapeutics.

[17] Xie F, Pullenayegum EM, Li SC (2010). Use of a disease-specific instrument in economic evaluations: Mapping WOMAC onto the EQ-5D utility index. Value in Health.

[18] Powell JL (1984). Least absolute deviations estimation for the censored regression model. Journal of Econometrics.

[19] Tobin J (1985). Estimation of relationships for limited dependent variables. Econometrica.

[20] Payakachat N, Summers KH, Pleil AM (2009). Predicting EQ-5D utility scores from the 25-item National Eye Institute Vision Function Questionnaire (NEI-VFQ 25) in patients with age-related macular degeneration. Quality of Life Research.

[21] Subramaniam M, Abdin E, Poon LY (2014). EQ-5D as a measure of programme outcome: Results from the Singapore early psychosis intervention programme. Psychiatry Research.

[22] Subramaniam M, Abdin E, Vaingankar JA (2013). Impact of psychiatric disorders and chronic physical conditions on health-related quality of life: Singapore Mental Health Study. Journal of Affective Disorders.

[23] Cheung YB, Luo N, Ng R (2014). Mapping the functional assessment of cancer therapy-breast (FACT-B) to the 5-level EuroQoL Group’s 5-dimension questionnaire (EQ-5D-5L) utility index in a multi-ethnic Asian population. Health and Quality of Life Outcomes.

[24] Wijeysundera HC, Tomlinson AJ, Norris CM (2011). Predicting EQ-5D utility scores from the Seattle Angina Questionnaire in Coronary Artery Disease: A mapping algorithm using a bayesian framework. Medical Decision Making.

[25] Austin PC (2002). A comparison of methods for analyzing health-related quality-of-life measures. Value in Health.

[26] Pullenayegum EM, Tarride JE, Xie F (2010). Analysis of health utility data when some subjects attain the upper bound of 1: Are Tobit and CLAD Models appropriate?. Value in Health.

[27] Baum CF (2006). An introduction to modern econometrics using stata.

[28] Sullivan PW, Ghushchyan V (2006). Mapping the EQ-5D index from the SF-12: US general population preferences in a nationally representative sample. Medical Decision Making.

[29] Chuang LH, Kind P (2009). Converting the SF-12 into the EQ-5D: An empirical comparison of methodologies. Pharmacoeconomics.

[30] Cheung YB, Tan LC, Lau PN (2008). Mapping the eight-item Parkinson’s Disease Questionnaire (PDQ-8) to the EQ-5D utility index. Quality of Life Research.

[31] Wang P, Luo N, Tai ES (2016). The EQ-5D-5L is more discriminative than the EQ-5D-3L in patients with diabetes in Singapore. Value in Health Regional Issues.

[32] Wailoo AJ, Hernandez-Alava M, Manca A (2017). Mapping to estimate health-state utility from non-preference-based outcome measures: An ISPOR good practices for outcomes research task force report. Value in Health.

[33] Verma S, Poon LY, Subramaniam M (2012). The Singapore Early Psychosis Intervention Programme (EPIP): A programme evaluation. The Asian Journal of Psychiatry.

[34] Verma S, Subramaniam M, Abdin E (2010). Safety and efficacy of long-acting injectable risperidone in patients with schizophrenia spectrum disorders: A 6-month open-lable trial in Asian patients. Human Psychopharmacology.

