# Supplementary material for: Mapping the Positive and Negative Syndrome Scale scores to EQ-5D-5L and SF-6D utility scores in patients with schizophrenia
Source: Qual Life Res. 2018 Oct 31;28(1):177–86. doi: 10.1007/s11136-018-2037-7 (PMC6339678; doi:10.1007/s11136-018-2037-7)
Supplement: Supplementary file 1 — Supplementary material 1 (DOCX 37 KB) [file 11136_2018_2037_MOESM1_ESM.docx]

| EQ-5D-5L | SF-6D |
| --- | --- |
|  |  |

Supplementary Figure 1. Histogram of residuals of EQ-5D-5L and SF-6D utility scores

Supplementary Table 1. Variance inflation factor of the final regression models for EQ-5D-5L and SF-6D utility scores

| EQ-5D-5L | | | SF-6D | | |
| --- | --- | --- | --- | --- | --- |
| Variable | VIF | 1/VIF | Variable | VIF | 1/VIF |
| Positive | 2.01 | 0.497150 | Positive | 1.53 | 0.652247 |
| Negative | 1.40 | 0.711863 | Depression | 1.40 | 0.712645 |
| General psychopathological symptoms | 2.53 | 0.394850 | Cognitive | 1.22 | 0.818290 |
| Female | 1.06 | 0.944969 | Negative | 1.17 | 0.853243 |
| Age | 1.00 | 0.998477 | Excitement | 1.21 | 0.828375 |
| Mean VIF | 1.60 |  | Mean VIF | 1.31 |  |
